# Analyzing influence of COVID-19 on crypto & financial markets and sentiment analysis using deep ensemble model

**DOI:** 10.1371/journal.pone.0286541

**Published:** 2023-09-28

**Authors:** Patrick Bernard Washington, Pradeep Gali, Furqan Rustam, Imran Ashraf

**Affiliations:** 1 Division of Business Administration and Economics, Morehouse College, Atlanta, GA, United Sates of America; 2 Department of Electrical Engineering, University of North Texas, Denton, TX, United States of America; 3 School of Computer Science, University College Dublin, Dublin, Ireland; 4 Information and Communication Engineering, Yeungnam University, Gyeongsan, Korea; Zayed University, UNITED ARAB EMIRATES

## Abstract

COVID-19 affected the world’s economy severely and increased the inflation rate in both developed and developing countries. COVID-19 also affected the financial markets and crypto markets significantly, however, some crypto markets flourished and touched their peak during the pandemic era. This study performs an analysis of the impact of COVID-19 on public opinion and sentiments regarding the financial markets and crypto markets. It conducts sentiment analysis on tweets related to financial markets and crypto markets posted during COVID-19 peak days. Using sentiment analysis, it investigates the people’s sentiments regarding investment in these markets during COVID-19. In addition, damage analysis in terms of market value is also carried out along with the worse time for financial and crypto markets. For analysis, the data is extracted from Twitter using the SNSscraper library. This study proposes a hybrid model called CNN-LSTM (convolutional neural network-long short-term memory model) for sentiment classification. CNN-LSTM outperforms with 0.89, and 0.92 F1 Scores for crypto and financial markets, respectively. Moreover, topic extraction from the tweets is also performed along with the sentiments related to each topic.

## Introduction

The COVID-19 pandemic left a substantial impact on the global economic market and several of the markets were shattered. It also damaged global markets and many industries like airline business, construction, and tourism industries recorded a large drop in shares [1]. Financial markets did not achieve their target because of the COVID-19 pandemic but during this period crypto markets grew very fast as BTC crossed the highest value of 65000$ during this time. Similarly, other cryptocurrencies also reached their highest values and gained the trust of the people during COVID-19. Consequently, a large number of research works were carried out to study the influence of COVID-19 on crypto markets and trend analysis of financial and crypto markets from an investment perspective during COVID-19 [2, 3].

The crypto and financial markets operate on distinct principles [4]. While the cryptocurrency market is decentralized, largely unregulated, and transparent, the traditional financial market is centralized, heavily regulated, and perceived to be more secure due to its established history [5]. The crypto market owns various digital assets that are traded using decentralized platforms such as Bitcoin and Ethereum. While the financial market works with various financial instruments, including stocks, bonds, commodities, and currencies [6]. These assets are traded on centralized exchanges, such as the New York Stock Exchange or the London Stock Exchange, and are subject to government regulations. However, these two markets are convoluted, and financial organizations are beginning to invest in crypto markets or provide crypto-related services to their customers. The surge in demand for cryptocurrencies has also prompted an increased demand for blockchain technology, which is now being utilized by financial market organizations to enhance their operations [7]. Investors assess the markets before deciding where to invest their resources using several tools. Tools make predictions about markets based on several factors, one of them being people’s sentiments about the market and which market is more in a talk on the internet.

Sentiment analysis has emerged as an important research area recently due to the wide use of social media platforms and the availability of a large amount of textual data on these networks. Sentiment analysis can be applied to determine the sentiments of people regarding specific events, persons, institutions, etc. Sentiment analysis techniques can be used for analyses of financial and crypto markets during COVID-19 as well. Twitter is a platform where people express their thoughts, opinions, and sentiments about different topics, products, and personalities [8, 9]. People discussed financial markets and crypto markets on Twitter which can be used for analysis between both markets. This study extracts the tweets dataset to find people’s sentiments about both financial and crypto markets during COVID-19 which can be used to get insight into future investments.

For sentiment analysis, machine learning models have been largely used as a manual analysis of sentiments is a laborious and time-consuming task. Machine learning models are suitable to process large amounts of data in small time and perform various preprocessing steps, feature extraction, and sentiment classification. This study proposes an approach for sentiment classification and data analysis using the supervised machine learning technique. We extract tweets that are posted by people during COVID-19 peak times using financial and crypto markets keywords. Preprocessing is carried out on the extracted data and an ensemble model is applied for sentiment classification. In brief, we make the following contributions which is a combination of convolutional neural networks and long-short terms memory (LSTM). We combined these models in stacked to perform the sentiment classification tasks. The contribution of this study is as follows:

- A large dataset is collected from Twitter regarding tweets related to financial and crypto markets during COVID-19.
- An ensemble deep learning model is proposed for sentiment classification with high accuracy. The ensemble mode incorporates a convolutional neural network (CNN) and a long short-term model (LSTM). The performance of the proposed model is compared with several machine learning and deep learning models. In addition, the efficacy of bag of words (BoW) and term frequency-inverse document frequency (TF-IDF) feature extraction approaches are evaluated.
- An analysis of financial and crypto markets during the COVID-19 period is also carried out where the financial market includes the stock market, bond market, and commodity market while for the crypto market Bitcoin, Ripple, Ethereum, and Dogecoin are considered.

This paper is further divided as follows. The Introduction section is followed by an overview of the literature about financial and crypto markets. Afterwards, description of the proposed approach, as well as, of the used models and dataset is provided. It is followed by the results and discussion while the study is concluded in the end.

## Literature review

Sentiment analysis has been a research focus in many areas such as politics, healthcare, product recommendations, and surveillance [10]. The sentiments of people, as expressed in their opinions, emotions, behavior, and feelings, significantly contribute to defining the investor’s opinions [11]. This section reviews comprehensive literature related to the underlying domain. To begin with, the application of sentiment analysis in financial markets is discussed, followed by its implementation for cryptocurrencies.

### Sentiment analysis for financial markets

The investment decisions are not entirely pragmatic, therefore, several studies attempted to understand the factors influencing the investment decision of an investor in financial and emerging markets. The study [12] investigated the trends in the Brazilian financial market by integrating sentiment analysis of related Twitter data. The tweets were assigned weight considering the three important factors including; a) retweets, b) favorites, and c) the total number of opinionated tweets. Moreover, the authors explored multiple time frames to evaluate the period in which the financial market is highly impacted by social media sentiments. Another study [13] forecasted the stock market prices for a time frame of 1 day, 7-days, 30-days, 60-days, and 90-days by exploiting the public sentiments using multilayer perceptron-artificial neural networks (MLP-ANN). The authors combined three datasets including Google trends, Twitter data, and News headlines, and concluded that increasing a variety of stock-related data enhances the performance of the classifier.

Likewise, the impact of sentiment analysis of Twitter data on the variation in the stock market was investigated using the Fuzzy decision platform in [14]. The authors utilized a lexical dictionary to extract the sentiments of Arabic tweets and achieved the highest accuracy score of 76%. Consequently, the study [15] stated that the emotions and sentiments extracted from stock-market-related data play a significant role in analyzing the trends in stock markets. The authors utilized the Syuzhet lexical dictionary for sentiment prediction and NRC lexical dictionary for emotion analysis. Similarly, the authors of [16] considered Pakistan, Turkey, Hong Kong, and US stock markets in addition to event sentiment for the prediction of the stock exchange. They explored the impact of local and global events by utilizing deep neural networks. The authors of [17] proposed a support vector machine (SVM) based sentiment analysis for the prediction of stock market trends. The study utilized TextBlob to extract the polarity score of each message. Experimental results revealed that optimizing the SVM’s parameters using particle swarm optimization (PSO) boosted its performance. In [18], the authors employed CNN, doc2vec, and long short-term memory (LSTM) to analyze the opinions posted on StockTwits. The study concluded that for the analysis of mass sentiment toward stock prices, sentiment analysis using CNN can be effective.

### Sentiment analysis for cryptocurrency

The authors of [19] investigated the association between Bitcoin (BTC) price fluctuation and tweets by integrating sentiment analysis. Based on the semantic aggregation, the proposed Naïve prediction model yielded 83% accuracy. In [20], the authors devised an extreme gradient boosting regression model (XGBoost) to predict the price fluctuation of cryptocurrency by investigating the sentiments of Twitter data. The tweets within the time frame of 3.5 weeks were collected and classified as positive, negative, and neutral. Furthermore, these tweets were given a sentiment indexing on an hourly basis resulting in weighted and unweighted tweets. Retweets were given a higher weight. The aforementioned six indices were utilized to train the proposed model with approximately 400 data points. Similarly, the study [21] utilized a modified valence-aware dictionary for sentiment reasoning (VADER), a cryptocurrency-specific lexical dictionary, to classify the sentiments of tweets related to nine cryptocurrencies. The authors suggested the price fluctuation of a variety of cryptocurrencies can be investigated by utilizing message volume and Twitter sentiment.

The study [22] stated that cryptocurrency-related Twitter data is a viable means of devising trading strategies. The authors employed several supervised machine-learning models to investigate the sentiments of the tweets. In line with this, another study [23] employed a deep neural network, LSTM, to classify the sentiments of crypto-related tweets to find an association between the price fluctuation of cryptocurrency and public perception. The authors constructed a lexical dictionary that is cryptocurrency-specific to extract the sentiments of the tweets. Consequently, the viability of the automated sentiment analysis of crypto-related tweets to acquire insights regarding the trading strategy was investigated in the study [24]. The authors employed tweets related to the altcoin cryptocurrency which were manually labeled as positive, negative, and neutral. A supervised machine learning model, random forest (RF) was employed to classify the tweet sentiments. The study stated that there is a significant association between the daily prices of cryptocurrencies and the number of tweets. Table 1 provides a comparative summary of the discussed works.

**Table 1:** Summary of the discussed research works.

Ref	Purpose	Proposed approach	Conclusion
[12]	Analysis of financial market trend in Brazil	MLP	The stock market is predictable using Twitter sentiments
[13]	Examining the correlation between stock market movement and social media content	MLP-ANN	Increasing the stock-related data improves the prediction accuracy.
[14]	Incorporating fuzzy decisions to enhance the decision-making of stock market investors.	Rule-based Fuzzy Decision	Utilizing fuzzy decisions into the system enhances the performance of the model.
[15]	Association between sentiment, emotions, and the stock market.	Naïve Bayes	The“Stock market” keyword can be utilized to predict the stock market movements with high accuracy.
[16]	Impact of local and global events on stock markets.	Convolutional Neural Network	Event sentiment analysis is proven to be effective in stock market prediction.
[17]	Stock market prediction based on sentiment analysis.	SVM + PSO	Sentiment analysis results in dimensionality reduction in addition to an improvement in accuracy by PSO.
[18]	Adaption of deep learning models for stock market prediction.	Convolutional Neural Network	The stock market can be analyzed with high efficacy using CNN.
[19]	Prediction of BTC’s price fluctuation.	VADER	A significant correlation exists between BTC price fluctuation and people’s sentiments.
[20]	Prediction of cryptocurrency price fluctuation using Sentiment analysis.	XGBoost	An analysis of trading volume and sentiments of tweets proposed model can predict price fluctuations within the cryptocurrency domain.
[21]	Estimating the predictive potential of Twitter sentiment for cryptocurrency’s price fluctuation.	VADER + Loughran & McDonald + Manually compiled Lexical Dictionary	Twitter is a viable source to investigate the price fluctuation of several cryptocurrencies.
[22]	Investigating BTC-related tweets using supervised machine learning models to devise a trading strategy.	Bernoulli Naïve Bayes (BNB)	Sentiment analysis of cryptocurrency-related Twitter data is effective in designing a trading strategy.
[23]	Exploit the correlation between Twitter data and price fluctuation in the crypto market.	LSTM + Lexical dictionary	Crypto sentiment analyzer provides highly correlated sentiments of crypto-related tweets resulting in high precision and recall by LSTM.
[24]	Investigating the relation between tweets and the daily process of cryptocurrency.	RF	Sentiment analysis of Twitter data proves to be an important source to analyze the price fluctuation of cryptocurrencies.

## Proposed methodology

**Fig 1:**
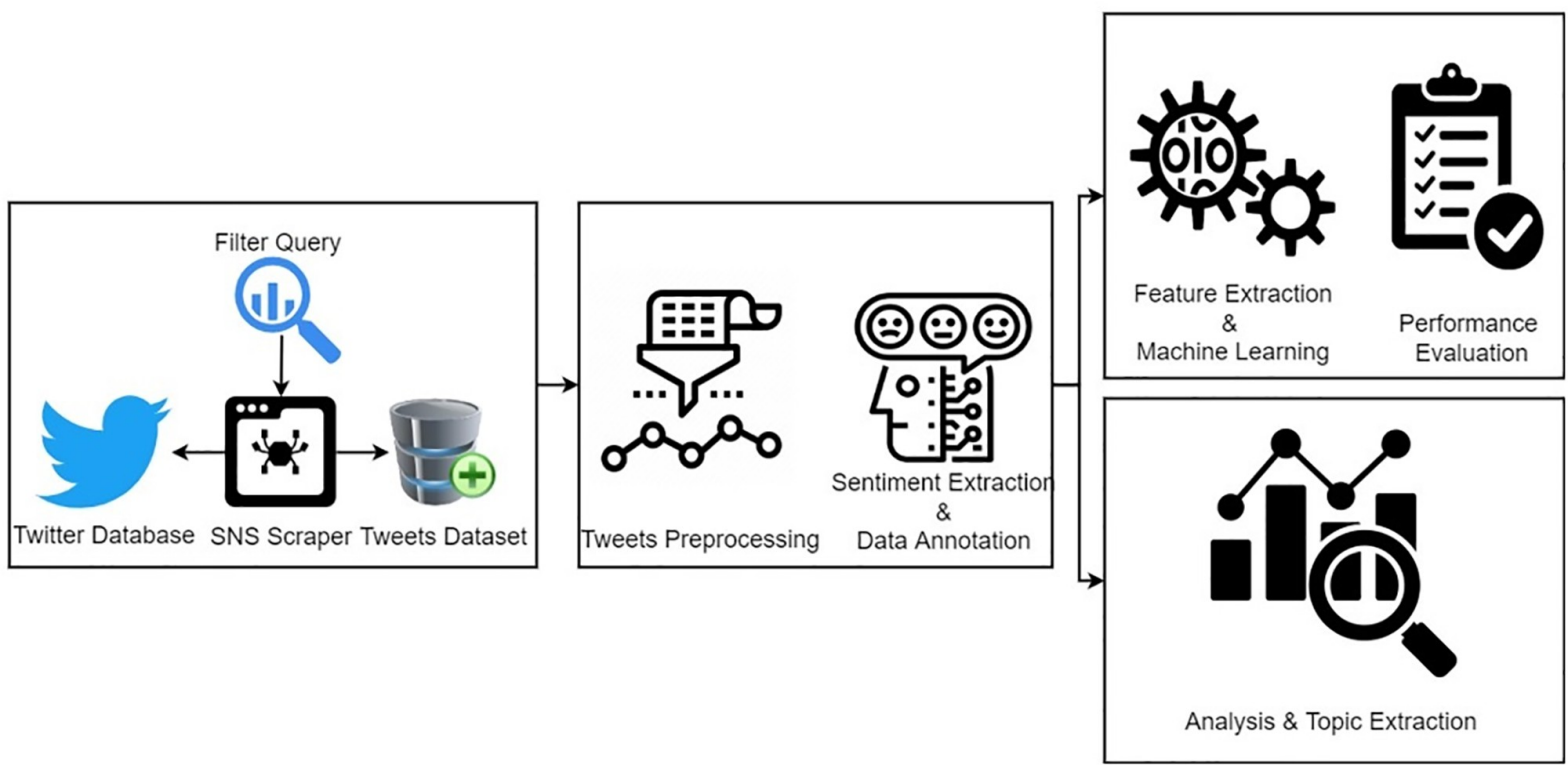
Workflow diagram of the adopted methodology.

This study proposes an ensemble model for sentiment classification regarding the analysis of financial and crypto markets. The workflow of the adopted methodology is shown in Fig 1.

To start with, we extracted tweets related to crypto markets and financial markets using the SNSscraper library. The extracted dataset in its raw form contains uniform resource locator (URLs), hashtags, and other meaningless information which we removed using several preprocessing steps. We used the hashtag, URLs, usernames removal technique, punctuation, and numeric removal technique, convert the text to lowercase, and performed stemming and stopword removal techniques. After preprocessing, we extract sentiment as positive, negative, and neutral for both markets using the TextBlob technique and also annotated both market tweets with these sentiments. After that, we performed analysis on both markets using the tweets dataset and extracted topics which are most discussed during COVID-19 for both markets and also analyzed the markets using Google trends. In the end, we proposed a machine learning approach for sentiment classification for both markets and in the machine learning approach, we used TF-IDF and BoW features and also deploy several machine learning and deep learning models for sentiment classification.

### Dataset description

This study performs sentiment analysis on crypto markets and financial markets during COVID-19. The data is extracted from Twitter using the social networking services (SNS) library SNSscraper using Python language. We extracted tweets from 2020–05-01 to 2021–05-01 regarding crypto markets and financial markets. We extracted a total of 40000 tweets; 20000 for each market. The complete dataset is publicly available at https://github.com/ImAshRayan/CryptoMarketAnalysis. The collection and analysis method complied with the terms and conditions of Twitter for tweet collection. Tweets id and contents are extracted in the dataset; a few samples of the dataset are shown in Table 2.

**Table 2:** A few samples from the dataset.

ID	Date	Tweet	Market
1.39E+18	2021–04–30 23:26:35+00:00	Just owning 3 ETFs allows you to hold the entire stock and bond market. Simple ETF Stragegy | simpleinvesting https://t.co/AJPFIkVad1	Financial
1.39E+18	2021–04–30 23:20:03+00:00	KRIS is the only default probability &amp; fixed income system which provides daily cross-validation https://t.co/Gy0guXng2X https://t.co/miwLb0nlH2	Financial
1.39E+18	2021–04–30 23:59:41+00:00	Bitcoin i have a possible bearish scenario so we need to watchout the market https://t.co/z4F9RgoPX4	Crypto

### Preprocessing

The text data generated in the real world is eminently exposed to noise, missing values, and disparity which highly affects the performance of machine learning classifiers. A variety of preprocessing techniques including tokenization, noise removal, Twitter features removal, case normalization, stemming, and lemmatization are considered within the scope of this study to acquire clean and structured data.

In preprocessing, tokenization is the segmentation of text into words, or phrases, namely tokens. Typically, alphanumeric or alphabetic characters separated by non-alphanumeric characters such as whitespaces, or punctuations are considered for text segmentation.

Tweets comprise usernames, URLs, and hashtags which are futile for the underlying analysis. We removed the tweet features from the underlying text data for future analysis. Noise removal aims to clean the text data of any irrelevant and redundant data from the underlying corpus. It involves the eradication of null values, numeric, punctuation marks, and stopwords (common words which are irrelevant to the analysis such as articles, prepositions, conjunctions, etc.) from the text data. Machine learning models are case sensitive [25]; therefore, we employed case normalization. The entire text data is transformed into lowercase before classification because there is no distinction between lowercase and uppercase forms of words.

Similarly, text data comprise a variety of inflected words which are semantically similar. This increases the dimensionality of the feature set which is addressed by stemming and lemmatization. Stemming truncates the prefixes and suffixes from the inflected words to transform them into their root forms [26]. On the contrary, lemmatization integrates the morphological analysis to covert the inflicted word into its base form or lemma [27]. A sample of the original tweet after employing preprocessing steps is shown in Table 3.

**Table 3:** A few samples from the dataset.

Original tweet	Preprocessed Tweet
@BTC_Archive: If #Bitcoin is speculative, what do you call a $200k student loan for a useless college degree? https://twitter.com/BTC_Archive/status/1471503640615538689	“speculative”, “call”, “student”, “loan”, “useless”, “college”, “degree”

### Sentiment extraction

Sentiment extraction of tweets utilizes a pre-defined lexical dictionary to provide the initial sentiment orientation of tweets in this study. A lexical dictionary is a database of words with their corresponding sentiment scores. This approach works by allocating respective sentiment scores to each token in the text segment, which is further aggregated into an overall polarity score *P*_*s*_. Each lexical dictionary has a polarity score range *R*_*p*_ which categorizes the text segment as positive, negative, or neutral based on the value of *P*_*s*_ within *R*_*p*_.

**Fig 2:**
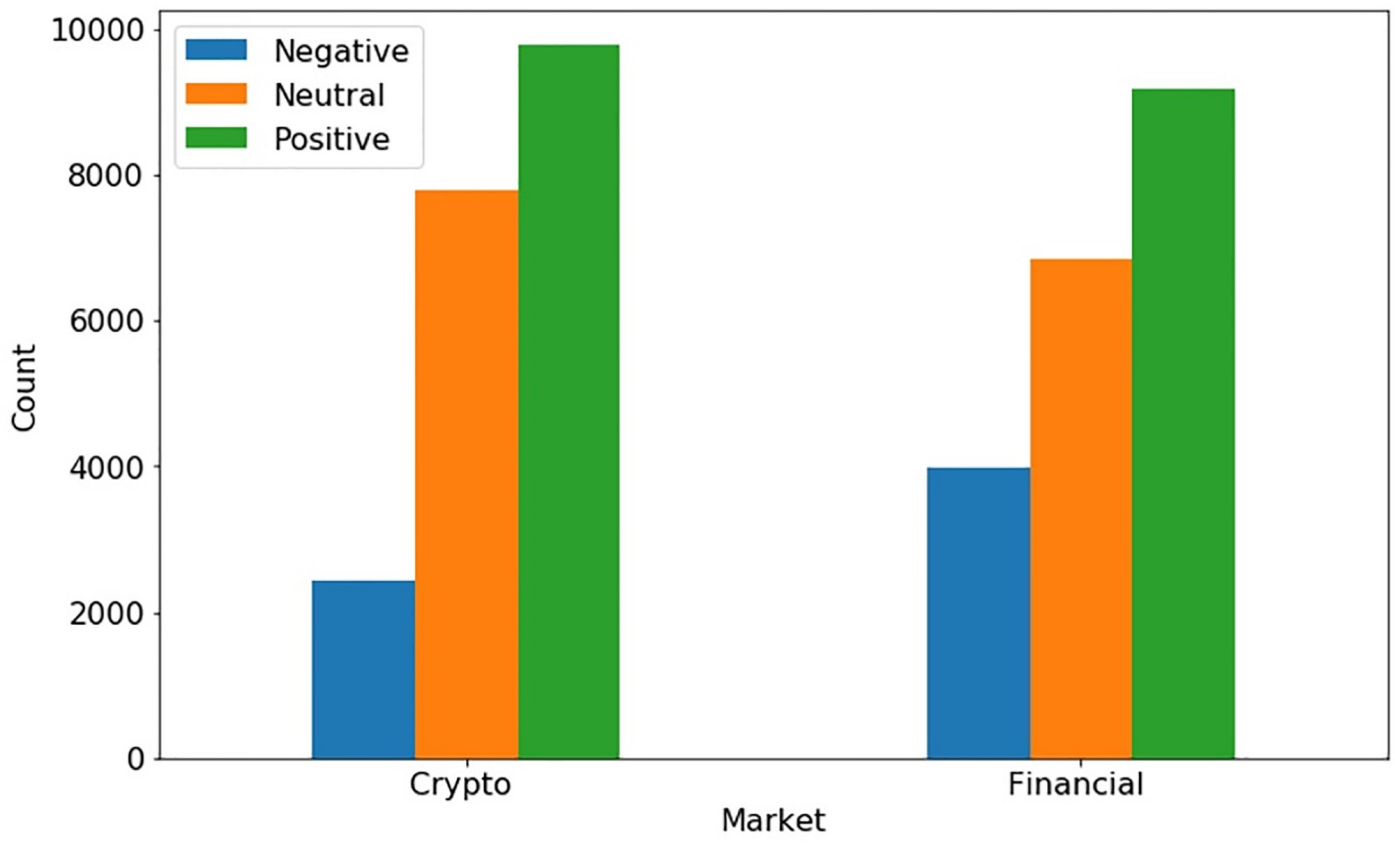
Sentiment count for both markets using TextBlob.

TextBlob is a well-known Python library that returns the polarity score of the underlying text data along with its subjectivity score [28]. The polarity score (*R*_*p*_) is considered in the underlying experiments and it ranges from +1 to -1, where, +1 refers to the positive sentiment, -1 refers to the negative sentiment, and 0 corresponds to the neutral sentiment. Sentiment counts for both crypto and financial markets are shown in Fig 2 using TextBlob.

### Feature extraction

The features are the vector representations of the raw data that can be utilized by machine learning algorithms to solve an underlying problem (i.e., classification). Unlike deep neural networks which can execute feature extraction on their own, the quality and quantity of extracted features highly impact the results generated by the machine learning algorithms [29]. Two well-known feature extraction techniques including TF-IDF and BoW are employed and compared in this study.

#### Term frequency—Inverse document frequency

TF-IDF is an information feature retrieval technique that quantifies the relevance of a word in a corpus. TF-IDF score of a word *w* contained in the tweet *t* from the dataset *D* is computed by taking a product of term frequency TF (frequency of occurrence of a word in a tweet) and inverse document frequency IDF (frequency of occurrence of a word in the entire dataset of tweets).

$$\begin{array}{c} TF-IDF(w,t,D)=tf(w,t)\times idf(w,d) \end{array}$$ (1)

where

$$\begin{array}{c} tf(w,t)=log(1+freq(w,t)) \end{array}$$ (2)

$$\begin{array}{c} idf\left(w,D\right)=log\frac{N}{count(t\in D:w\in t)} \end{array}$$ (3)

#### Bag of words

The BoW is a simple and flexible text representation technique that quantifies a word in the given dataset. It disregards any information related to the syntax, and significance of a word in the dataset thus referred to as a “bag” of words. BoW primarily involves two metrics; a dictionary of known words and the frequency of occurrence of known words in the entire dataset. This feature extraction technique considers each word as a feature. The BoW features indicate a significant milestone in sentiment analysis of tweets [30].

### Machine learning models

Sentiment classifiers learn the input set of vectors in correspondence to the known target values and classify unseen instances. Several sentiment classifiers including RF, LR, DT, and SVM are considered in the underlying study to carry out predictive tasks. Each classifier is used with its optimized parameter settings which are selected based on its prevalent use in the field of sentiment analysis as shown in Table 4. A detailed description of each classifier is given here.

**Table 4:** Model hyperparameters settings.

Model	Hyper-parameters	Tuning Range
RF	n_estimators = 300, max_depth = 300	n_estimators ={20 to 500}, max_depth={20 to 500}
DT	max_depth = 300	max_depth={20 to 500}
LR	solver = liblinear, C = 3.0	solver = liblinear, C={1 to 5}
SVM	Kernel = linear, C = 3.0	Kernel =[rbf,linear], C={1 to 5}

DT is a supervised classifier that has a flow-chart-like tree structure. DT is constructed and trained recursively. It chooses the pre-eminent set of features that are further utilized to split the input vectors [28].

RF is a tree-based supervised machine learning classifier that constructs an ensemble of DTs to reduce the variance and enhance predictive accuracy. Each DT is trained on the random sample of train data by integrating the aggregated bootstrap technique [31].

LR is a statistical machine learning classifier that integrates the sigmoid function ‘*σ*’ to directly model the probabilistic ratio. *σ* is an ‘S-shaped curve that restricts the predictive value within the given range. Moreover, it offers insights regarding the significance of the predictor [32].

SVM is a supervised learning algorithm that can be utilized for classification and regression tasks. The SVM algorithm plots each input vector as a data point in the *k*-dimensional vector space (*k* = total number of input vectors). The predictive task is performed by allocating an optimum hyper-plane that discriminated between the target values [33].

### Topic extraction

Since the text data comprises words, a topic that is discussed in several tweets can be interpreted by combining a group of highly associated words. Each tweet is considered to be composed of a variety of topics that can be inferred using topic extraction techniques. It represents each tweet as a diverse set of interrelated topics and each topic as a diverse set of interrelated words. The underlying study performs topic extraction by integrating Latent Dirichlet allocation (LDA), which is considered to show optimum results among several other topic extraction techniques [34].

LDA considers each tweet as a collection of probabilistically distributed words or topics that are extracted based on the probability of overlapping words in it. LDA considers two fundamental metrics; a) the dataset is comprised of topics, and b) topics are comprised of words. The number of topics is defined prior to its implementation. Latent in LDA refers to the hidden topics, Dirichlet is the distribution of topics in the dataset of tweets, and allocation refers to the topic extraction once the Dirichlet is determined.

Statistically, LDA defines the documents as a probability distribution of topics *θ*(*td*), and topics as the probability distribution of words *ϕ*(*wt*)

$$\begin{array}{c} \theta (td)=P(t|d) \end{array}$$ (4)

$$\begin{array}{c} \phi (wt)=P(w|t) \end{array}$$ (5)

The probability of a word in a given document containing topics *T* is computed as

$$\begin{array}{c} \sum_{t\in T}p\left(w|t\right),d)p\left(t|d\right) \end{array}$$ (6)

### Proposed ensemble model

**Fig 3:**
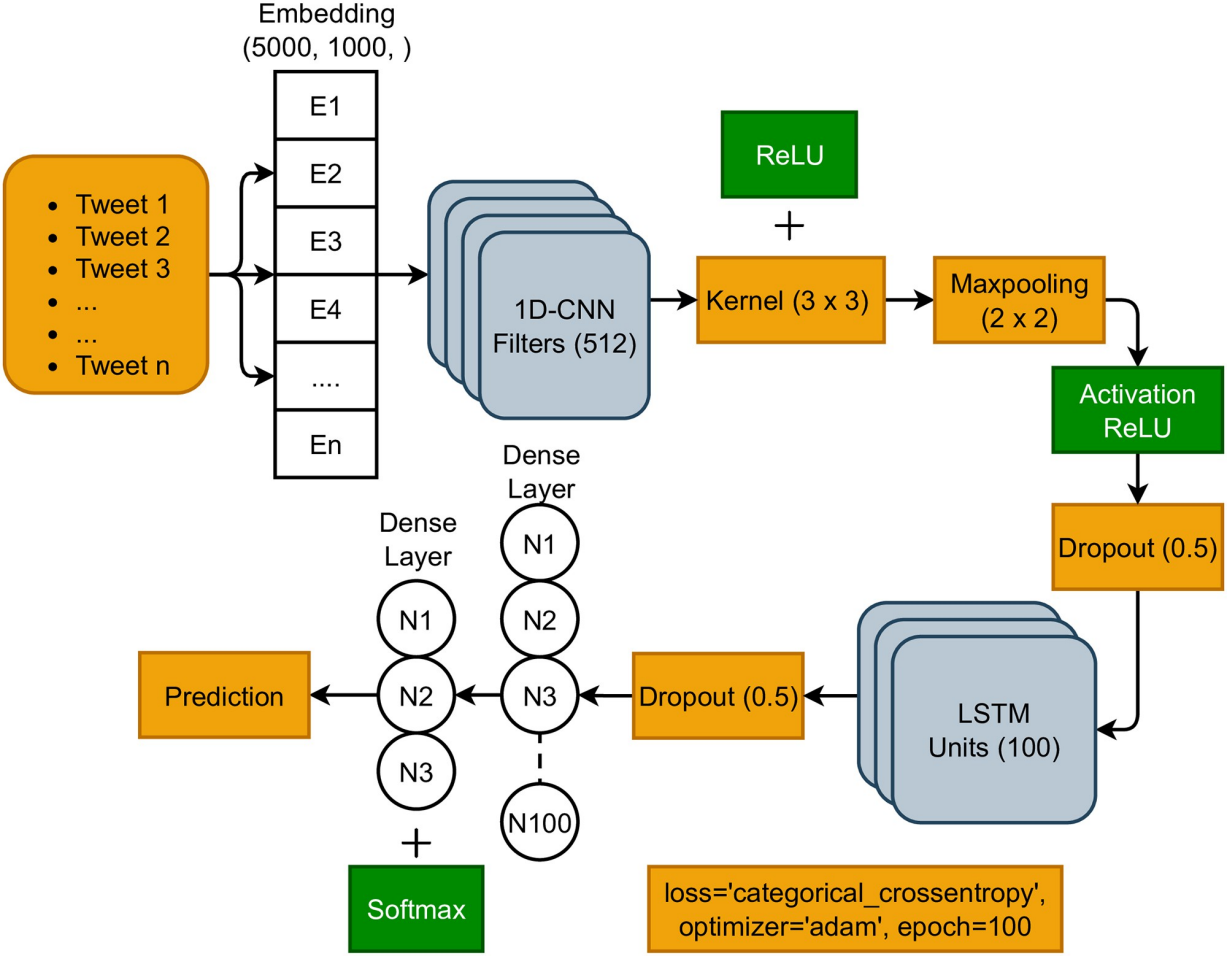
Architecture of the proposed model.

This study proposes an ensemble model for sentiment classification using state-of-the-art CNN and LSTM models. The CNN model is specially designed for image data and LSTM for text data but in this study, we used text classification in combination [35, 36]. These models are combined sequentially as on the top CNN extracts the feature and LSTM uses that feature to make the prediction. We select both models because CNN is good for large data to extract worthy features using convolutional architecture, and LSTM is better for text data because of its recurrent architecture [37]. We used an embedding layer on the top with a 5000 vocabulary size and 1000 output dimensions. This embedding layer will take text data as input and generate a numeric representation for CNN. The 1-dimensional (1D) CNN layer with 512 filters and 3 × 3 kernel size is used after the embedding layer. Here, the number of output channels after convolution, while the size of the convolution filter used to perform convolution on the image is referred to as the kernel [38]. This CNN layer is followed by the max-pooling layer with a pool size of 2 which helps to extract a worthy feature set of convoluted data. Rectified linear unit (ReLU) is used with the activation function layer after max-pooling which helps to create non-linearly in the network. A dropout layer with a 0.2 dropout rate is used after that to reduce the complexity of the data [39]. The LSTM layer with 100 units is followed by the dropout layer. After the LSTM layer, again dropout layer and a dense layer is used. In the end, the proposed model is compiled with the ‘Adam’ optimizer, ‘categorical_crossentropy’ loss function, and 100 epochs. Fig 3 shows the proposed model architecture.

## Results and discussions

This section contains the sentiment analysis results on crypto and financial markets during the COVID-19 period. We analyze the tweets related to these markets and find the topics which were mostly used in extracted tweets and the sentiments of people on those topics. We also discuss the results of the machine learning approach for sentiment classification for crypto and financial markets. We deployed the proposed approach on the Corei7 7th generation system with Windows OS. We used sci-kit learn, SNSscraper, Tensorflow, and NLTK library and implemented the proposed methodology on Jupyter Notebook in Python language.

### Analysis on crypto and financial markets using topic modeling and Google trends

**Fig 4:**
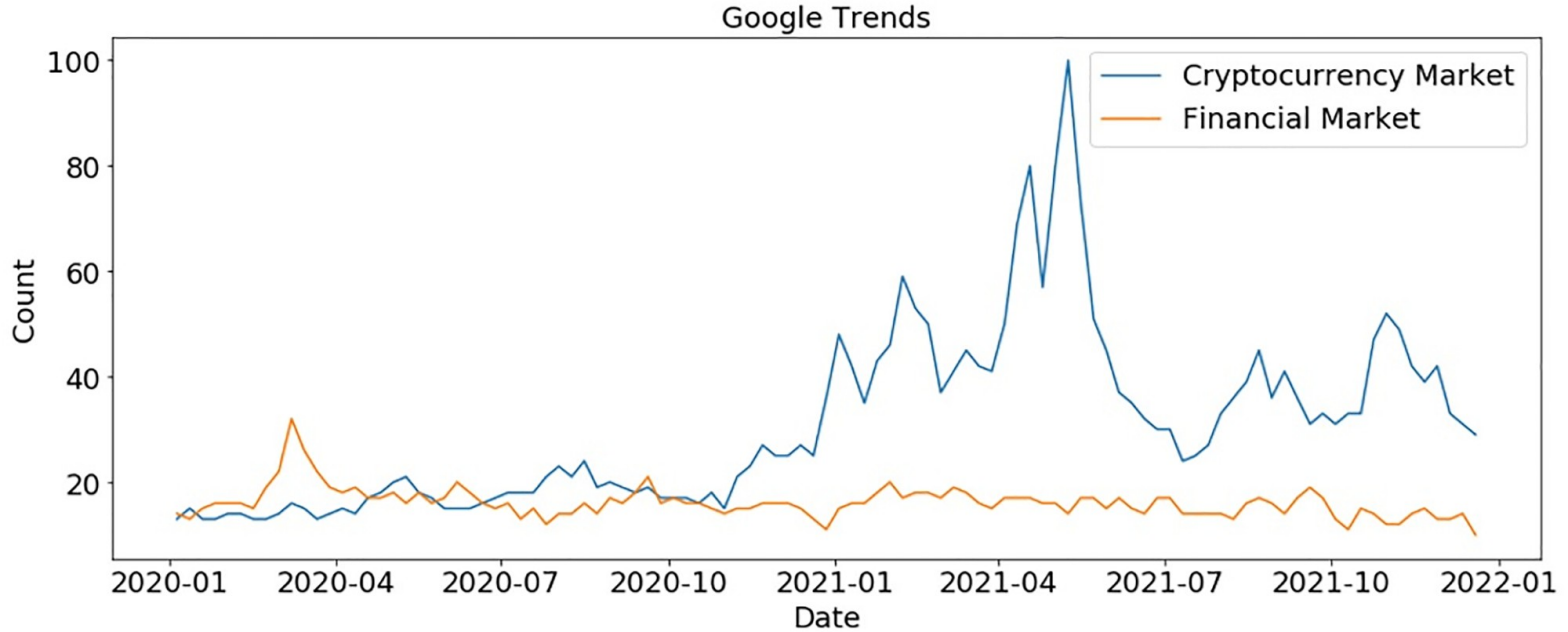
Google trends for crypto market vs financial market.

We perform analysis using Google trends data to show people’s interest in both crypto and financial markets. Fig 4 shows that people show more interest in crypto markets during COVID-19 as compared to financial markets. There is a little higher interest at the start of 2020 for the financial market as compared to the crypto market but later, the crypto market gained more interest at the start of 2021 because during the COVID-19 stock market crashed but crypto markets witnessed its peak as BTC touches the highest values. As COVID-19 became largely under control during the mid of 2021, people’s interest in the crypto market was reduced but still, it had higher attraction than the financial market.

**Fig 5:**
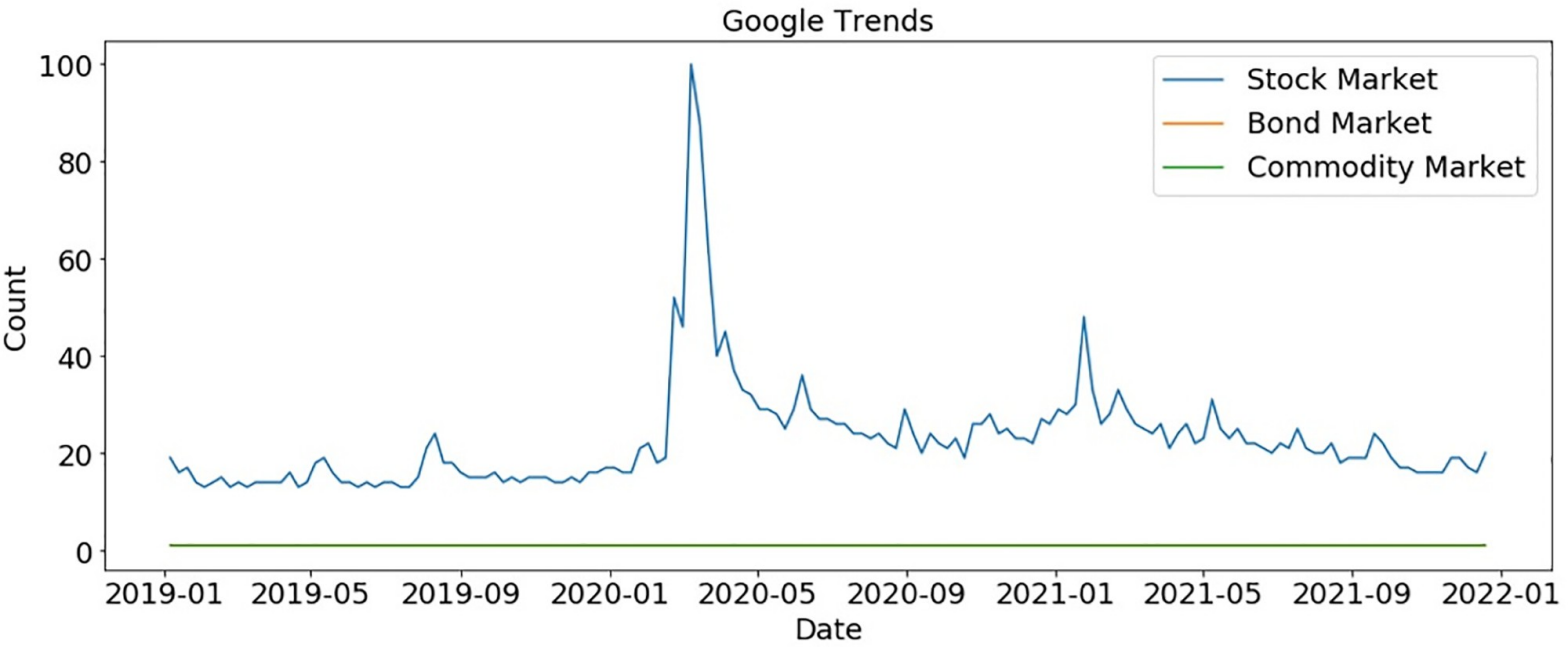
Google trends for the financial market.

According to Fig 4, people show more interest in financial markets at the start of 2020 so we further explore which finical markets gained interest at the start of 2020. We found that the stock market gained more interest as compared to other financial markets such as bond markets and commodity markets, as shown in Fig 5. But this polarity suddenly goes down as COVID-19 cases increased worldwide.

**Fig 6:**
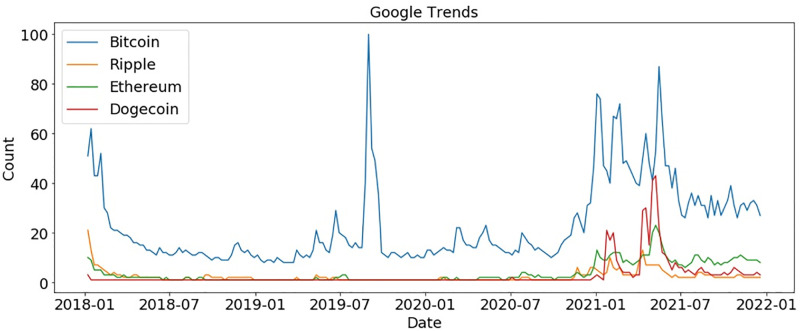
Google trends for the crypto market.

We also explored the crypto market further and analyzed which currency gained more interest in crypto markets during COVID-19. Fig 6 shows that Bitcoin and Dogecoin gained more interest during the COVID-19 period, respectively. Four major cryptocurrencies Bitcoin, Ripple, Ethereum, and Dogecoin received higher market value during COVID-19. The analyses show that the crypto market gets more interest from people as compared to financial markets during COVID-19.

### Most discussed topics on twitter related to crypto and financial markets during COVID-19

**Fig 7:**
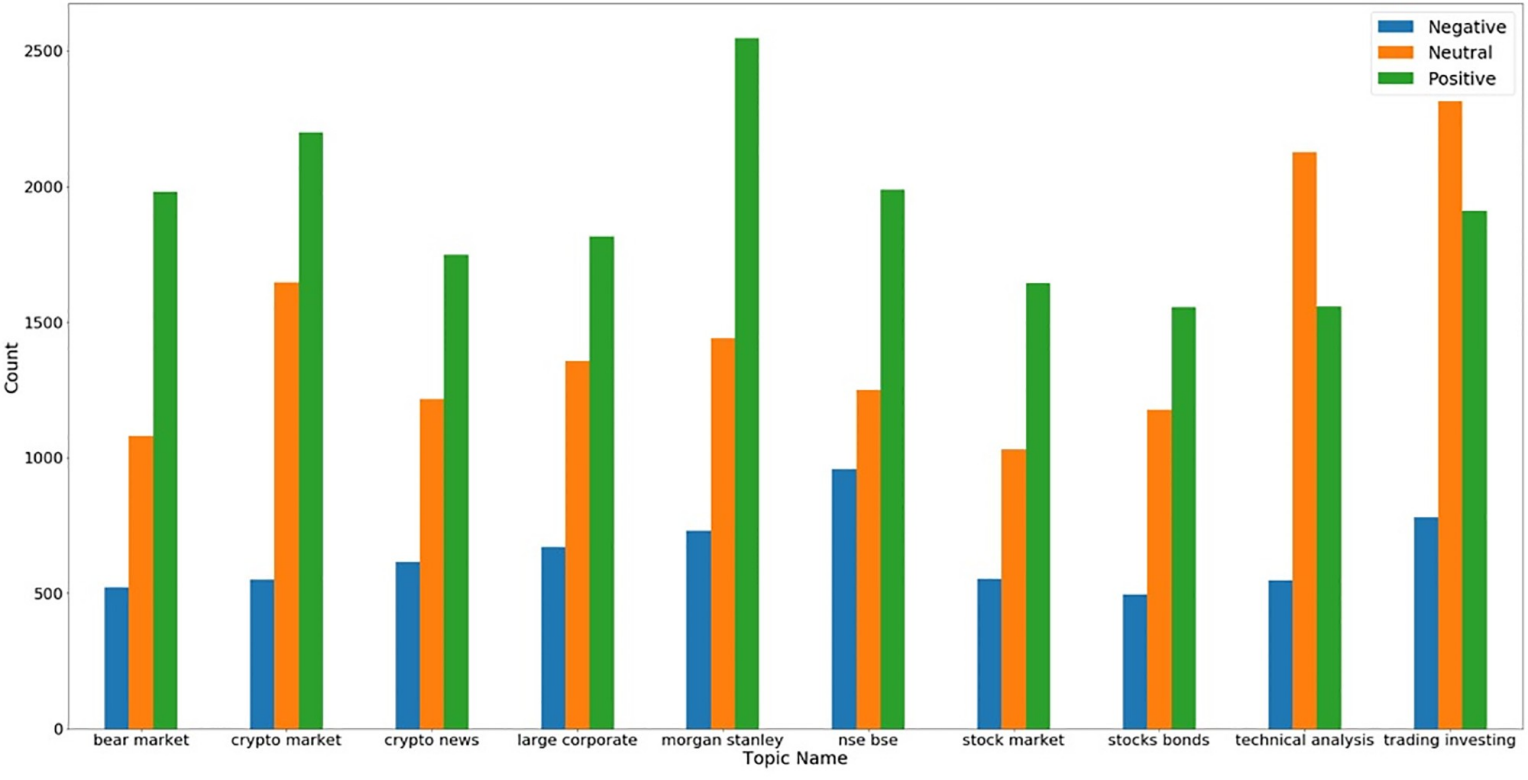
Top 10 topics in the extracted dataset and their corresponding sentiments.

We extracted the most discussed tweets in our dataset and selected the top 10 topics. We also performed sentiment analysis on each topic and found people’s sentiments on that topic. Fig 7 shows the extracted topics from the overall dataset and their corresponding sentiments. Topics are related to the stock market, crypto market, and bond market. The ratio of positive sentiments is more as compared to negative in all the topics. Most tweets during COVID-19 discussed Morgan Stanley which a well-known investment banking company and the sentiment of people are mostly positive for Morgan Stanley. On the other hand, national stock exchange (NSE) and BSE-related tweets show more negative sentiment of people in comparison to other topics which shows the crash of India’s NSE during COVID-19.

**Fig 8:**
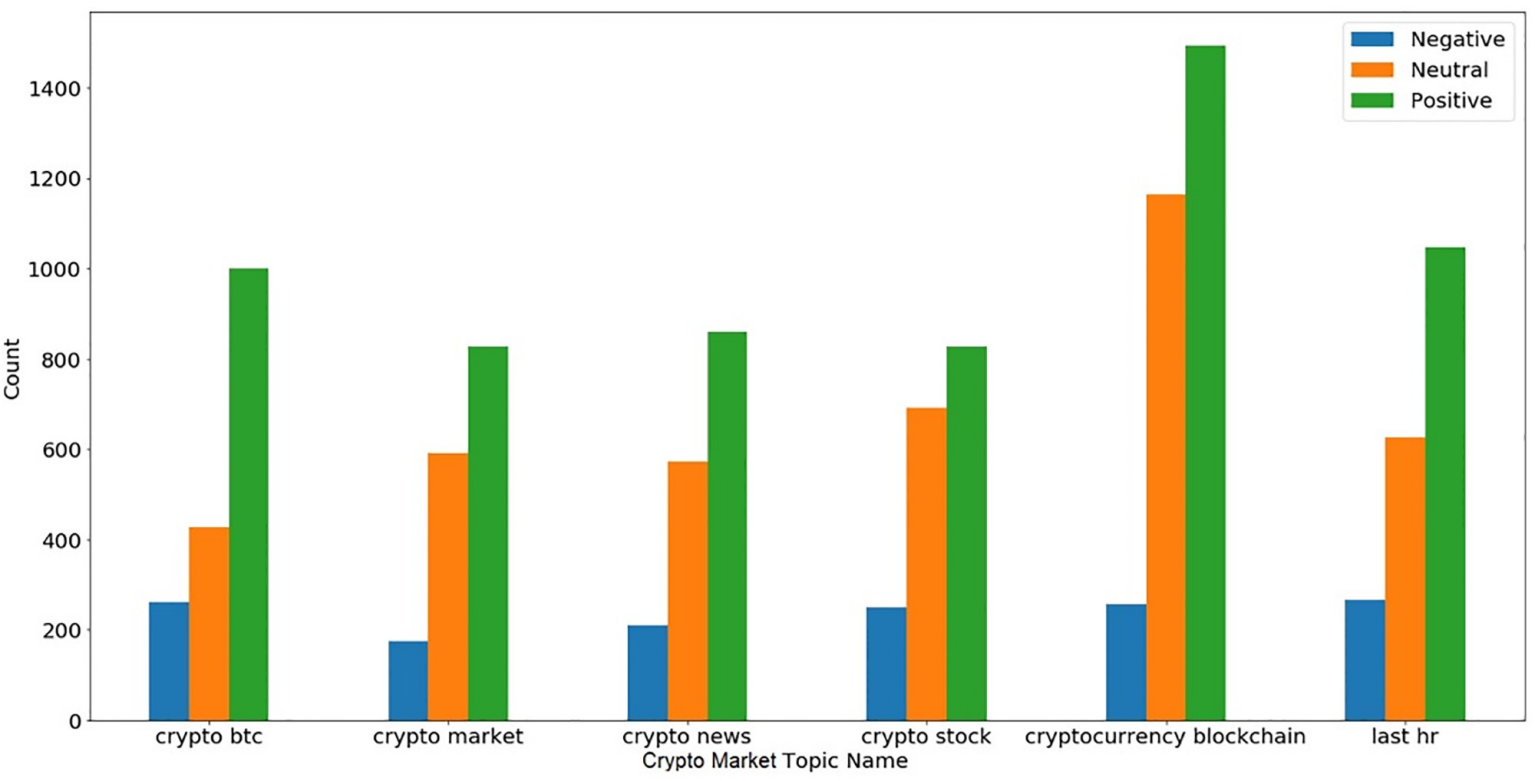
Top 10 topics in the crypto market-related tweets dataset and their corresponding sentiments.

**Fig 9:**
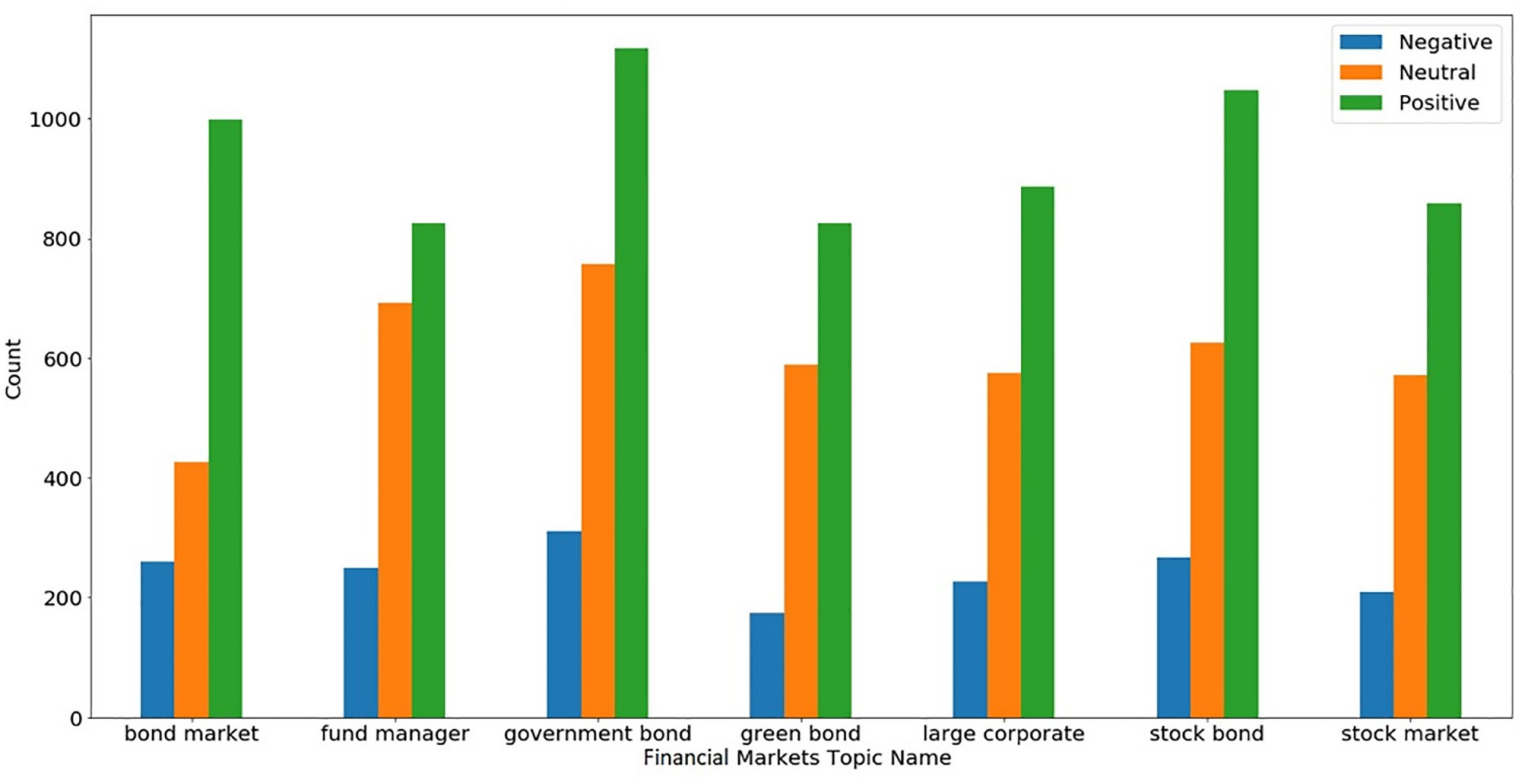
Top 10 topics in the crypto market-related tweets dataset and their corresponding sentiments.

Fig 7 contains the topics from the whole dataset while Figs 8 and 9 show topics separately for the crypto market and financial markets. According to Fig 9, most tweets discuss crypto blockchain, and this topic gains more positive sentiments. One of the interesting topics is the last hr in crypto-related tweets which means that updates of the crypto market for the last hour are very popular and we can conclude that from this topic last hour index values are very important in crypto markets for investment decisions.

Fig 9 shows the topics for financial markets during the COVID-19 period and people’s sentiments for all topics are mostly positive. Government bonds get more positive sentiment as compared to others and other topics like green bonds and fund managers are more interesting. Overall analysis shows that topics and tweets contain more positive sentiment as compared to negative for both crypto and financial markets but the interest of people was more towards the crypto markets as compared to financial markets.

### Performance of models for sentiment classification on crypto and financial markets

This section contains the results of machine learning models for sentiment classification. We deploy these models with BoW and TF-IDF features and for classification, we used Textblob annotated dataset.

#### Sentiment classification results for crypto market

Results for machine learning models with BoW and TF-IDF features are shown in Table 5. The performance of all models is good for crypto market data as SVM achieved the highest 0.93 accuracy with a 0.88 F1 score. This significant performance of SVM is because of the large text feature set. SVM and LR both are good for the large feature set and show better performance in this study as well due to a large feature set.

**Table 5:** Performance of machine learning models on crypto market data.

Feature	Model	Accuracy	Precision	Recall	F1 score
BoW	SVM	0.93	0.89	0.87	0.88
	LR	0.92	0.90	0.85	0.87
	DT	0.84	0.81	0.72	0.73
	RF	0.89	0.91	0.76	0.79
TF-IDF	SVM	0.90	0.89	0.82	0.84
	LR	0.90	0.89	0.80	0.83
	DT	0.83	0.79	0.72	0.74
	RF	0.88	0.89	0.74	0.77

Table 6 contains the result of deep learning models. It can be observed that the results of the proposed CNN-LSTM model are better crypto data and the results of deep learning models are more significant as compared to the machine learning models. LSTM and CNN-LSTM both achieved the highest accuracy 0.94 but the proposed model exceeds the LSTM in terms of F1 score. Since the dataset is imbalanced, F1 is given more importance for this study. The proposed model is significant as compared to all other models with a 0.89 F1 Score.

**Table 6:** Performance of deep learning models on crypto market data.

Model	Accuracy	Precision	Recall	F1 score
LSTM	0.94	0.90	0.86	0.88
CNN	0.93	0.89	0.87	0.88
CNN-LSTM	0.94	0.90	0.88	0.89

#### Sentiment classification results for financial market

Sentiment classification results for financial market data using machine learning models are shown in Table 7. The results of machine learning models are better on the financial market dataset as compared to the crypto market dataset. The better performance is due to financial data being less imbalanced as compared to crypto data. SVM and LR show significant performance in comparison with other models as SVM achieved a 0.93 accuracy score with a 0.91 F1 score on financial data and LR obtained a 0.92 accuracy score and 0.90 F1 score.

**Table 7:** Performance of machine learning models for financial market data.

Feature	Model	Accuracy	Precision	Recall	F1 score
BoW	SVM	0.93	0.92	0.91	0.91
	LR	0.92	0.92	0.89	0.90
	DT	0.83	0.85	0.79	0.80
	RF	0.88	0.90	0.84	0.86
TF-IDF	SVM	0.91	0.91	0.88	0.89
	LR	0.90	0.90	0.87	0.88
	DT	0.82	0.83	0.78	0.79
	RF	0.87	0.89	0.82	0.84

Experimental results on financial data using deep learning models are shown in Table 8. Results indicate that the CNN-LSTM model achieved the highest F1 score compared to all other used models. This significant performance of the proposed models is because of its ensemble architecture. Features extracted using CNN tend to increase the performance of LSTM and the overall performance of the ensemble model is improved significantly.

**Table 8:** Performance of deep learning models for financial crypto market data.

Model	Accuracy	Precision	Recall	F1 score
LSTM	0.93	0.91	0.91	0.91
CNN	0.92	0.90	0.90	0.90
CNN-LSTM	0.93	0.92	0.91	0.92

### Discussions

**Fig 10:**
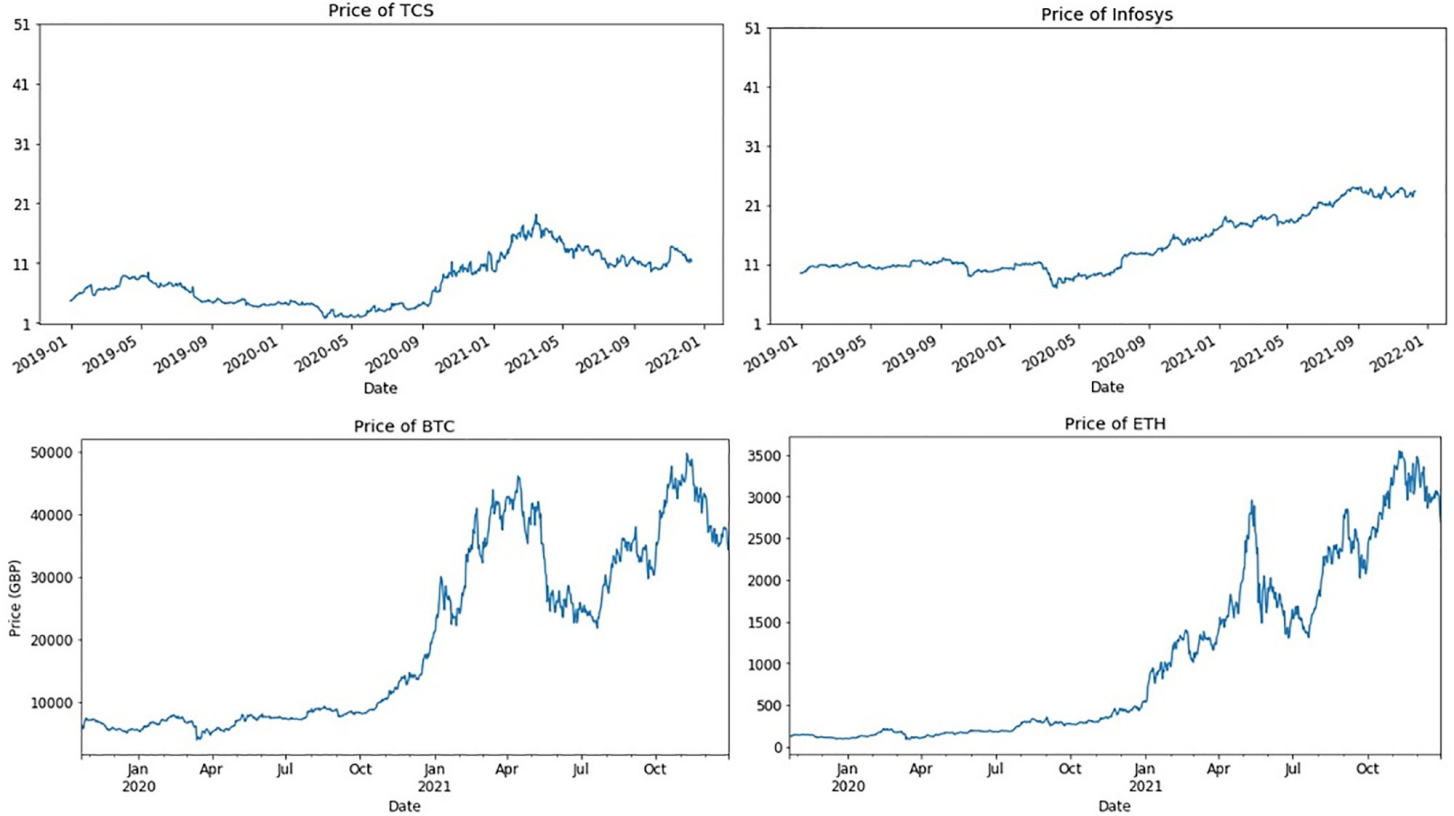
Comparison in real values of crypto and financial markets.

In this study, we performed sentiment analysis on crypto and financial markets-related tweets posted during COVID-19. We analyzed which market gained more interest from the people and what were the sentiments of people during that time regarding investments. According to analysis, people took more interest in crypto markets as compared to financial markets. People have more positive sentiments towards crypto markets as compared to financial markets. In addition, the search on the interest was high for crypto markets. Also, higher investments are made in crypto markets as compared to financial markets. Consequently, the crypto market observed a large increase in market value. Market values of two cryptocurrencies Bitcoin and Ethereum and two financial markets Infosys and Tata Consultancy Service are shown in Fig 10. According to values, the BTC and ETH markets get a huge increase in price while Infosys and TCS could not gain such an increase. We also proposed an approach for sentiment classification for both crypto and financial markets using deep learning models which perform significantly on tweets datasets.

### Analysis after 10 months gap according to Google trends

**Fig 11:**
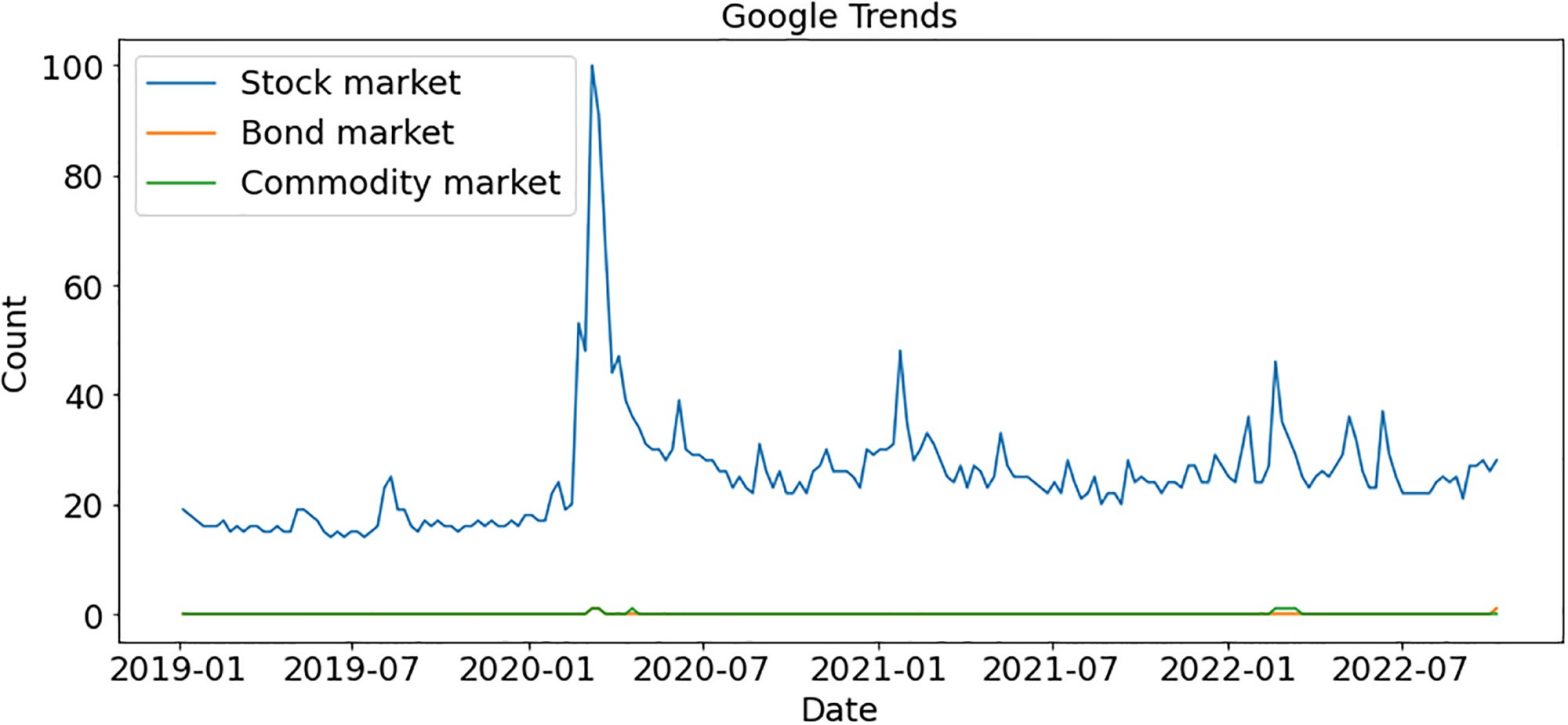
Comparison of markets after 10 months according to Google trends.

**Fig 12:**
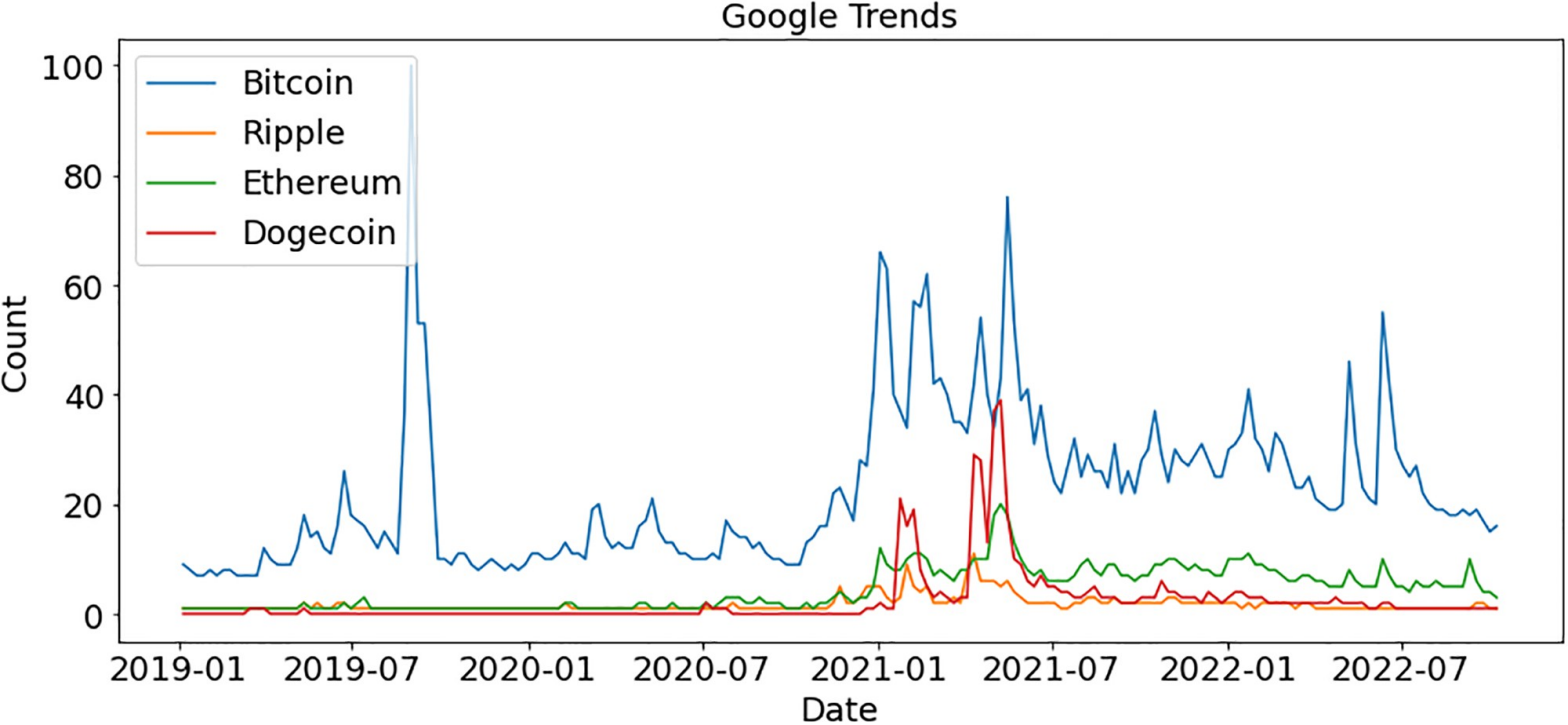
Comparison of cryptocurrency after 10 months according to Google trends.

We analyzed the same topics after a 10-month gap to determine if trends had changed. The peak moment for the stock market was during COVID-19, at the start of 2020. As shown in Fig 11, people showed no interest in any market after that period. During the crypto market bubble in 2019, Bitcoin reached a peak, and then again at the start of 2021, Bitcoin gained popularity. Bitcoin has ups and downs regarding people’s interests, whereas other currencies cannot get traders’ attention, as shown in Fig 12.

### Comparison with previous studies

This section presents a comparative analysis of the proposed approach and other recent studies in the same sentiment analysis domain. Since the collected dataset is unique, there are no previous studies on this dataset. To ensure a fair comparison, we implement the recent studies and approaches on our dataset. Specifically, we selected studies that work in the same domain and deploy their approaches in the same environment as our approach. For instance, Aslam et al. [40] proposed a hybrid deep learning approach for sentiment analysis of cryptocurrency tweets using a recurrent architecture LSTM-GRU, which achieved significant accuracy. Huang et al. [41] used a simple LSTM architecture for sentiment classification, while Hasan et al. [42] employed chi-squared (chi2) features with RF. The chi2 feature selection technique was used to select important features, and RF was used for sentiment classification. We implement these studies separately on both financial and crypto market datasets, and the comparison results are presented in Table 9.

**Table 9:** Comparison with recent studies.

Study	Year	Model	Market	Accuracy	Precision	Recall	F1 Score
[40]	2022	LSTM-GRU	Financial	0.86	0.85	0.84	0.85
			Crypto	0.88	0.86	0.86	0.86
[41]	2021	LSTM	Financial	0.87	0.85	0.85	0.85
			Crypto	0.87	0.79	0.78	0.79
[42]	2022	RF	Financial	0.87	0.88	0.86	0.87
			Crypto	0.88	0.85	0.84	0.84
Proposed	2023	LSTM-CNN	Financial	0.94	0.90	0.88	0.89
			Crypto	0.93	0.92	0.91	0.92

### Statistical T-test

To show the significance of the proposed approach, we performed the statistical T-test, which finds the statistical difference between two compared results [43]. T-test rejects or accepts the null hypothesis (*N*_*H*_) on the basis of compared results; if the compared results are statistically different, then T-test rejects *N*_*H*_ otherwise accepts *N*_*H*_. The *N*_*H*_ can evaluate different output scores such as T-statistic (T), critical value (CV), and p-value (P). We find the results with T-test by using the alpha value 0.5, also known as the significance level. For rejection of *N*_*H*_, there are two scenarios,

$$\begin{array}{c} \left(abs\left(T\right)\leq CV\right):\;\;N_{H}\left(1\right) \end{array}$$ (7)

$$\begin{array}{c} \left(P<Alpha\right):\;\;N_{H}\left(2\right) \end{array}$$ (8)

If the results of the T-test satisfies the above consideration then it rejects the *N*_*H*_ which means compared results are statistically significant. Table 10 shows the results of the T-test and according to the results and almost all cases *N*_*H*_ is rejected while only in two cases is accepted when we compared results with SVM with BoW features. These results show that the proposed approach is statistically significant as compared to other used methods.

**Table 10:** Statistical t-test results for compared approaches.

Market	Case	T	CV	P	*N*_*H*_ (1)	*N*_*H*_ (2)
Crypto	CNN-LSTM Vs. SVM	-0.538	0.00	0.61	Reject	Accept
	CNN-LSTM Vs. LR	-0.859	0.00	0.423	Reject	Reject
	CNN-LSTM Vs. DT	-4.464	0.00	0.004	Reject	Reject
	CNN-LSTM Vs. RF	-1.764	0.00	0.128	Reject	Reject
	CNN-LSTM Vs. LSTM	-0.348	0.00	0.740	Reject	Accept
	CNN-LSTM Vs. CNN	-0.538	0.00	0.610	Reject	Accept
Financial	CNN-LSTM Vs. SVM	-0.397	0.00	0.705	Reject	Accept
	CNN-LSTM Vs. LR	-1.464	0.00	0.194	Reject	Reject
	CNN-LSTM Vs. DT	-7.137	0.00	0.00	Reject	Reject
	CNN-LSTM Vs. RF	-3.693	0.00	0.010	Reject	Reject
	CNN-LSTM Vs. LSTM	-0.775	0.00	0.468	Reject	Reject
	CNN-LSTM Vs. CNN	-2.324	0.00	0.059	Reject	Reject

## Conclusion

Crypto and financial markets are big investment platforms but during COVID-19 some markets inflicted high losses on investors. The analysis performed in this study shows that the crypto markets were at a peak during the COVID-19 period. There are no big ups and downs in people’s interest in financial markets according to Google Trends. We also found that the real values of crypto markets increase much faster during COVID-19 while the financial markets could not gain much worth. For sentiment classification, we deployed the deep learning and machine learning approach and showed that deep learning can be more accurate as compared to machine learning models. We deployed a hybrid deep learning approach in which we combined CNN and LSTM. Experimental results indicate that the combination of models can perform better as compared to individual models as it achieved significant 0.94 and 0.93 accuracy scores for Crypto and Financial markets, respectively. In future work, we intend to perform an analysis of both markets by incorporating additional data from other social media platforms such as Facebook, Instagram, etc. We also intend to add financial analysis regarding crypto and financial markets.

## Supporting information

S1 File (IPYNB)

S2 File (IPYNB)

S3 File (CSV)

S4 File (CSV)

S5 File (CSV)

S6 File (CSV)
